# The Nature and Extent of Online Marketing by Big Food and Big Alcohol During the COVID-19 Pandemic in Australia: Content Analysis Study

**DOI:** 10.2196/25202

**Published:** 2021-03-12

**Authors:** Florentine Martino, Ruby Brooks, Jennifer Browne, Nicholas Carah, Christina Zorbas, Kirstan Corben, Emma Saleeba, Jane Martin, Anna Peeters, Kathryn Backholer

**Affiliations:** 1 Deakin University Geelong Australia, Global Obesity Centre Institute for Health Transformation; 2 School of Communication and Arts The University of Queensland Brisbane Australia; 3 Victorian Health Promotion Foundation Melbourne Australia; 4 Obesity Policy Coalition Melbourne Australia

**Keywords:** alcohol, food and beverage, COVID-19, marketing, social media

## Abstract

**Background:**

Emerging evidence demonstrates that obesity is associated with a higher risk of COVID-19 morbidity and mortality. Excessive alcohol consumption and “comfort eating” as coping mechanisms during times of high stress have been shown to further exacerbate mental and physical ill-health. Global examples suggest that unhealthy food and alcohol brands and companies are using the COVID-19 pandemic to further market their products. However, there has been no systematic, in-depth analysis of how “Big Food” and “Big Alcohol” are capitalizing on the COVID-19 pandemic to market their products and brands.

**Objective:**

We aimed to quantify the extent and nature of online marketing by alcohol and unhealthy food and beverage companies during the COVID-19 pandemic in Australia.

**Methods:**

We conducted a content analysis of all COVID-19-related social media posts made by leading alcohol and unhealthy food and beverage brands (n=42) and their parent companies (n=12) over a 4-month period (February to May 2020) during the COVID-19 pandemic in Australia.

**Results:**

Nearly 80% of included brands and all parent companies posted content related to COVID-19 during the 4-month period. Quick service restaurants (QSRs), food and alcohol delivery companies, alcohol brands, and bottle shops were the most active in posting COVID-19-related content. The most common themes for COVID-19-related marketing were *isolation activities* and *community support*. Promotion of hygiene and home delivery was also common, particularly for QSRs and alcohol and food delivery companies. Parent companies were more likely to post about corporate social responsibility (CSR) initiatives, such as donations of money and products, and to offer health advice.

**Conclusions:**

This is the first study to show that Big Food and Big Alcohol are incessantly marketing their products and brands on social media platforms using themes related to COVID-19, such as *isolation activities* and *community support*. Parent companies are frequently posting about CSR initiatives, such as donations of money and products, thereby creating a fertile environment to loosen current regulation or resist further industry regulation. “COVID-washing” by large alcohol brands, food and beverage brands, and their parent companies is both common and concerning. The need for comprehensive regulations to restrict unhealthy food and alcohol marketing, as recommended by the World Health Organization, is particularly acute in the COVID-19 context and is urgently required to “build back better” in a post-COVID-19 world.

## Introduction

In March 2020, the World Health Organization declared a global COVID-19 pandemic, which is caused by the virus SARS-CoV-2 [1]. People over the age of 60 years and those with underlying noncommunicable diseases, such as cancer, obesity, cardiovascular disease, and diabetes, are at higher risk of developing severe illness from COVID-19 [2]. These conditions impair the immune system and the ability to fight off viruses [3]. Consumption of alcohol and ultraprocessed foods are leading risk factors for these underlying conditions [4]. Additionally, heavy alcohol consumption, both short and long term, is known for its direct immunosuppressive effects [5].

The COVID-19 pandemic is also having serious consequences for mental health. A recent poll demonstrated that the pandemic has negatively impacted the mental health of 48% of parents and 36% of children in Australia [6]. Well-known coping mechanisms for dealing with stress and negative emotions include excessive consumption of alcohol [7,8] and overeating of unhealthy, highly palatable foods [9]. Indeed, evidence shows that population consumption of unhealthy foods and excessive alcohol intake increases after mass traumas, such as global financial crises, terrorism, and natural disasters [7,10,11]. This may, in turn, exacerbate mental and physical ill-health. For example, alcohol use is associated with increased reporting of interpersonal and domestic violence incidents [12]. Similarly, unhealthy diets are associated with poor mental health outcomes for adults and children, such as depression [13] as well as anxiety and mood disorders [14]. Building healthy populations resilient to the ongoing threat of COVID-19 and potential future public health threats will require a concerted effort to reduce obesity, unhealthy dietary intake, and excessive alcohol consumption across the population [15].

Marketing of unhealthy foods and beverages and alcohol influences attitudes, preferences, expectations, and consumption of these products over the life course [16]. With the rise of social media and other digital platforms, marketing is omnipresent, increasingly targeted, immersive, and engaging [17]. Consumption of alcohol and unhealthy foods and beverages is frequently portrayed by the alcohol and food industries as fun and harmless [18,19]. Global examples of marketing by these industries during the COVID-19 pandemic suggest that unhealthy food and alcohol brands and companies are capitalizing on the pandemic to promote their products, market corporate social responsibility (CSR), pursue partnerships, and shape policy environments [20]. The alignment of brands and companies with a social or health issue, known as *cause marketing*, is a subset of CSR business practices [21]. Cause marketing is used to build goodwill and to enhance public perceptions of the brand [22]. Social media platforms are ideal platforms to promote CSR activities, as interaction with consumers is high, maximizing opportunities to expand audience reach [23]. Regardless of whether or not cause marketing has positive social impacts, it is counterproductive if companies promote products that contribute to the problems they purport to solve. For example, alcohol brands have been found to “pinkwash” their products by aligning themselves with the pink ribbon of the breast cancer awareness campaign, which is paradoxical, as alcohol is an established risk factor for breast cancer [24].

No study to date has systematically and comprehensively examined if “Big Alcohol” and “Big Food” are capitalizing on the COVID-19 pandemic to increase sales by “COVID-washing” their online posts. This study aimed to examine the nature and extent of COVID-19-related online posts by leading alcohol and unhealthy food and beverage brands and their parent companies in Australia over 4 months (February to May 2020) during the COVID-19 pandemic.

## Methods

### Study Design

Similar to previous studies that investigated marketing by Big Food and Big Alcohol on social media [25,26], we conducted a content analysis of the public accounts on social media platforms of leading alcohol brands, unhealthy (ie, energy-dense and nutrient-poor) food and beverage brands, and their parent companies. Content analysis is “a research technique for making replicable and valid inferences from texts (or other meaningful matter) to the contexts of their use” [27]. All COVID-19-related posts were retrospectively extracted in June 2020 for a 4-month time period during the first wave of the COVID-19 pandemic in Australia, with data collected between February 1 and May 31, 2020. During these 4 months, various actions to limit the spread of COVID-19 were implemented in Australia, including public health orders to stay at home. As this research only involved analysis of publicly available social media accounts, institutional ethics approval was not required.

### Selection of Brands and Companies

We selected the top five brands in Australia and their parent companies across the following categories: (1) confectionery, (2) snacks, (3) soft drinks, (4) quick service restaurants (QSRs), (5) food delivery services, (6) beer, (7) wine, (8) spirits, (9) ready-to-drink (RTD) alcoholic beverages, (10) alcohol retailers, and (11) alcohol delivery services (see Multimedia Appendix 1). These Australian brands were selected based on Australian market share (ie, sales) [28].

Some of the top five of RTD alcoholic beverage and spirit brands overlapped (eg, Jim Beam had an RTD alcoholic beverage and a spirit in the top five), and some of the top five bottle shops and alcohol delivery companies overlapped (eg, Dan Murphy’s was in top five of both categories). We combined these categories when presenting results. Brands that did not sell ultraprocessed foods and beverages according to the NOVA classification [29], such as dairy brands, were excluded and replaced by the brand with the next largest market share.

### Selection of Social Media Platforms

We selected brands’ and companies’ official public accounts on the following social media platforms, where available: Facebook, Instagram, YouTube, and Twitter. These four social media platforms were chosen for the following reasons:

1. Facebook, Instagram, and YouTube are the most frequently used platforms by Australians, and Twitter is an important platform for public relations communication from major brands and corporations.
2. These platforms are commonly used by large brands and companies for both advertising and public relations.
3. Facebook, including its subsidiary Instagram, and Google, including its subsidiary YouTube, constitute an advertising duopoly. The Australian Consumer and Competition Commission estimates that Facebook and Google receive two-thirds of the online advertising spend in Australia [30].

We sampled the social media accounts that were most likely to target Australian audiences. Therefore, only the Australian accounts of brands were included. Parent companies, on the other hand, are often global entities with multiple brands in their portfolio and often did not have a local social media presence in Australia. If this was the case, international accounts were selected, where available. Occasionally, brands and parent companies used the same accounts (eg, Arnott’s, Lindt, and Uber Eats), in which case the data were only captured in the *brands* analysis. Finally, we excluded social media accounts that had not been active during the prior 12 months (ie, since February 1, 2019).

### Data Collection

For each social media platform, we collected data on the number of followers, the total number of posts, and details of COVID-19-related posts that brands and companies shared using their public social media accounts. The date of these posts, the product marketed, and number of likes, views, and shares—at least one week after sharing the post—were recorded in a standardized template.

**Figure 1:**
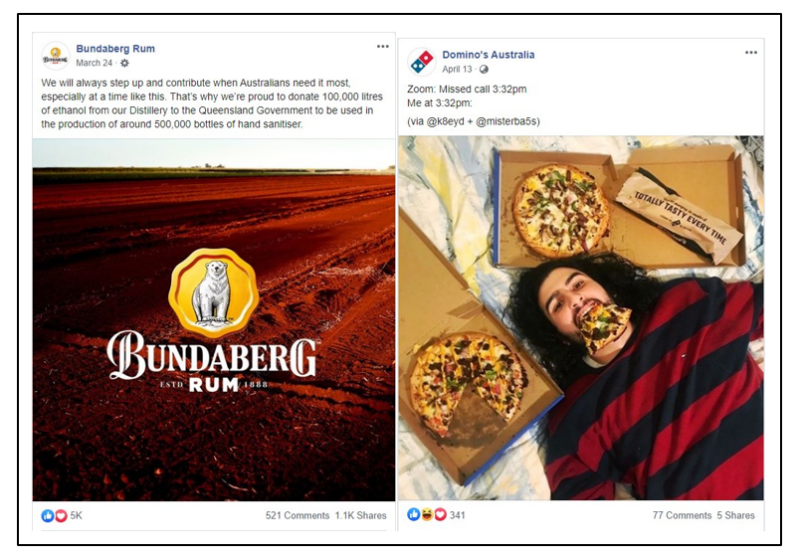
Examples of posts directly (left) and indirectly (right) related to the COVID-19 context.

COVID-19-related posts were included if they directly or indirectly referred to the COVID-19 pandemic or issues pertaining to COVID-19 (see Figure 1), such as lockdown and social distancing. Indirect marketing included posts where the only relationship to COVID-19 was the addition of hashtags (eg, #workfromhome, #quarantinecooking, and #quarantini).

Only official brand- and company-generated posts on their social media accounts or channels were included in our analysis. For Facebook, we collected posts and reposts, including photos, videos, and events; for Instagram, we collected posts, pinned stories, and hashtag promotions in account bios; for YouTube, we collected videos; and finally, for Twitter, we collected tweets and retweets. We did not include user-generated content. For all four social media platforms, we collected the number of followers or channel subscribers (see Table 1).

**Table 1:** Social media followers by platform and Australian brand account during the first week of May 2020.

Brands		Followers on social media platforms, n			
		Facebook	Instagram	YouTube	Twitter
**Confectionery**					
	Cadbury	16,704,000	24,500	3250	15,100
	Allen’s	378,000	11,500	513	<100
	Darrell Lea	128,000	14,600	<100	1000
	Lindt	7,196,000	14,000	1030	N/A^a^
**Snacks**					
	Arnott’s	93,700	13,900	3350	2800
	Peters	42,200	N/A	267	N/A
	Magnum	12,412,000	N/A	3400	N/A
	Smith’s	161,900	600	3400	N/A
**Soft drinks**					
	Coca-Cola	1,059,000	45,000	7450	6200
	Pepsi Max	37,511,000	1200	2990	800
	Schweppes	85,000	4100	367	1200
	Kirks	130,000	200	<100	N/A
**Quick service restaurants**					
	McDonald’s	80,537,000	156,000	22,900	32,800
	KFC	55,241,000	154,000	9510	29,600
	Hungry Jack’s	664,000	61,200	4600	11,800
	Domino’s Pizza	1,126,000	97,400	4120	37,700
	Subway	809,600	18,100	2460	9300
**Food delivery services**					
	Uber Eats	2,767,000	49,700	N/A	N/A
	Deliveroo	751,000	21,100	<100	600
	DoorDash	N/A	N/A	N/A	N/A
	Menulog	118,000	6800	1350	3700
**Beer**					
	XXXX Gold	426,000	13,200	31,500	300
	Corona Extra	15,119,000	34,800	15,800	700
	Carlton Premium Dry	191,000	17,600	2140	900
	Victoria Bitter	154,000	33,000	1100	1900
	Great Northern Original Lager	121,000	31,900	800	N/A
**Spirits and ready-to-drink alcoholic beverages**					
	Smirnoff Red	14,168,000	6800	440	300
	Johnnie Walker Red Label	14,901,000	N/A	1410	N/A
	Jim Beam	3,205,000	N/A	600	300
	Jack Daniel’s	17,304,000	9000	1060	5700
	Bundaberg	372,000	22,200	1640	1700
	Canadian Club	283,000	12,900	200	700
	Woodstock & Cola	60,100	2000	<100	N/A
**Wine**					
	De Bortoli	20,000	10,000	306	8200
	McWilliam’s	14,800	12,700	<100	2700
	Stanley Wines	N/A	N/A	N/A	N/A
	Jacob’s Creek	853,000	27,200	1430	5000
	Berri Estates	<100	N/A	N/A	N/A
**Bottle shops and alcohol delivery services**					
	BWS	269,000	11,600	N/A	N/A
	Liquorland	152,000	3400	<100	1400
	Dan Murphy’s	477,000	42,500	6850	9300
	Cellarbrations	60,200	1000	<100	200
	IGA Plus Liquor	13,600	N/A	N/A	N/A
	Shop My Local	<100	<100	N/A	N/A
	Jimmy Brings	16,400	5179	Not displayed^b^	200

^a^N/A: not applicable; the brand did not have an Australian social media account or had not posted since February 2019 and was excluded from the analysis.

^b^This brand did have an active platform, YouTube in this case; however, the number of subscribers was not displayed. This account was included in the analysis.

### Data Analysis

We developed a coding framework based on an initial analysis of COVID-19-related posts by a subset of brands in our sample. Three investigators identified COVID-19-related themes used in social media posts by the top brand and company in each category. These initial themes were discussed with the research team in order to develop a coding framework that could be applied across all sampled social media accounts. The framework continued to develop iteratively as additional themes were identified in the data (see Table 2).

Three researchers extracted and coded the social media posts. Every individual post was independently extracted and coded by one researcher (RB or JB), and a subsample (20%) of all posts by the top brands in every product category was cross-checked for consistency of data extraction and coding by a third researcher (FM). Discrepancies in data extraction and coding were discussed between researchers and, where necessary, through consultation with a fourth researcher (KB) until consensus was reached. Because there were very few discrepancies in coding, we did not continue this process for the remaining brands. Similarly, any unclear posts and how these should be coded were discussed between the three coders and the senior member of the research team (KB) throughout the analysis.

Once all the COVID-19-related posts had been identified and coded according to the framework, we calculated the frequency with which each COVID-19-related theme was used by each brand and company and across product categories (see Table 2).

**Table 2:** COVID-19-related marketing themes.

COVID-19 theme	Description	Example
Trading or event updates	Practical updates around trading hours, opening or closing of stores, and events during the COVID-19 pandemic, excluding delivery	“We’re still open! As you’ve just heard from our PM, no restrictions will apply to off-licence venues. So take your time, visit us in-store or online 7 days a week. #danmurphys” (Dan Murphy’s—Twitter, local brand)
Home delivery or take away in lockdown period	Mentions of home delivery or take away options during the COVID-19 pandemic (eg, no need to leave the house and staying at home during difficult times)	“Working from home? You can still enjoy your Macca’s faves and have them dropped on your doorstep with contactless McDelivery. We’ll deliver to you.” (McDonald’s—Instagram, local brand)
Hygiene or zero contact	References to reducing chances of virus spread through hygiene practices when preparing food or handling food and drinks, social distancing by employees and customers, and extra cleaning and disinfecting (eg, contactless, zero contact, and keeping community safe)	“We won’t let anything get in the way of you and your sub, not even a little <-- social distancing --> Come see us in-restaurant. We’re still open for takeaway -- just make sure you stay about five Subway Footlong® subs apart” (Subway—Facebook, local brand)
Community support or feeling	References to standing together during these challenging, unprecedented, or unexpected times and/or the brand or company being there to support consumers and we are all in this together	“Connect with your mates online and we’ll get through this together, with a GOLD in hand” (XXXX Gold—Facebook, local brand)
Applauding health staff or essential workers	Referencing or thanking essential workers, health workers, frontline workers, etc, including discounts for these workers	“The Philippines continues to be under strict lock down due to the coronavirus, making it difficult for many communities to provide for their families. Our frontline healthcare workers are overworked as we overcome this pandemic. To help share comfort and nutrition, #TeamMDLZ #Philippines has shared P12MM worth of snack products to communities and frontline healthcare workers, working with 40+ organizations. #StrongerTogether” (Mondelez—Facebook, international parent company)
Donations	References to large-scale product or financial donation	“Beam Suntory and Southern Glazer’s Wine & Spirits $1 million donation will provide resources and financial aid to workers affected by mandated closures amidst the COVID-19 pandemic” (Beam Suntory—Facebook, international parent company)
Isolation activities	Suggestions for things to do while in isolation that include brand use or promotion	“#StayHome and get your bake on this week with this tantalising TeeVee Snacks Caramel Slice! A deliciously indulgent snack that will really hit the sweet spot. https://bit.ly/3bqGIxd” (Arnott’s—Facebook, local brand)
Consumption helps with coping with COVID-19	References to consumption making consumers feel better or to consumers deserving the advertised product	“Jump on our site to organise drinks for your staff in 3 simple steps. Then jump on Zoom and crack a cold one together - a bit of normalcy is great for team morale!” (Jimmy Brings—Instagram, local brand)
Supporting local business or trading partners	Suggests consumers should support local businesses, or announcements that the brand or company supports local businesses or their trading partners	“This is a very challenging time for many of our customers. As part of our ongoing support, Coca-Cola Amatil has established a free 24-hour customer support and counselling service for our customers who are struggling with the unprecedented impact of COVID-19. The Coca-Cola Amatil Customer Support Line is run by Assure, a trusted Amatil partner. The 24/7 support service offers confidential counselling and financial coaching and is available to all Amatil customers completely free of charge, in the strictest confidence. Contact your Coca-Cola Amatil representative for all the details.” (Coca-Cola Amatil—Facebook, local parent company)
Health advice, including mental health	Posts include health, including mental health, advice with reference to COVID-19	“Touching multiple surfaces, then our faces, is one of the most common causes of infection. Find out how bleach can clean and protect your home from our expert Suresh Nadakatti.” (Unilever—Twitter, international parent company)
Production of sanitizing products	References to the brand or company making hand sanitizer	“#ICYMI: Brown-Forman and others in the spirits industry convert distilling operations to produce sanitizer to combat the COVID-19 health crisis. https://bddy.me/2y3FNEb” (Brown Forman—Twitter, international parent company)
Maintaining an essential product supply chain	References to supply chains being maintained, ensuring consumers’ needs are met	“Our nine factories across Australia and New Zealand are hard at work making product. Our dedicated teams are doing their best to bring your favourite Nestlé food and beverage products to a store near you. Look after yourself and each other out there! ❤️ Nestlé” (Nestlé—Facebook, international parent company)

## Results

### Food and Alcohol Brands

After excluding brands and services without Australian accounts in Australia, 42 brands were included in the analysis. Table 3 shows that most brands had low or no activity on YouTube and Twitter (<20 posts over 4 months), with a few exceptions such as Jimmy Brings on YouTube (24 posts; 0% COVID-19 related) and Domino’s on Twitter (24 posts; 38% [9/24] COVID-19 related). The number of account followers (see Table 1) shows that brands generally have the greatest social media followings on Facebook and Instagram. For example, McDonald’s Australia had 80,537,000 followers on Facebook, 156,000 on Instagram, 23,000 on YouTube, and 33,000 on Twitter.

Most brands (33/42, 79%) posted content that related to the COVID-19 pandemic. A total of 916 COVID-19-related posts out of 2796 posts (32.8%) were identified. Engagement by followers in terms of likes, shares, and views was substantial and varied greatly between brands and type of content (see Table 3), with more active accounts generating more interactions, in general, as did videos regardless of platform. The product categories with the highest proportions of total posts related to COVID-19 were bottle shops and alcohol delivery (231/619, 37.3%), QSRs (353/915, 38.6%), food delivery (52/142, 36.6%), beer (86/226, 38.1%), spirits and RTD alcoholic beverages (34/121, 28.1%), and wine (83/261, 31.8%) (see Table 3). COVID-19-related marketing activity was very low or nonexistent for brands within the snacks and soft drink categories. Arnott’s was the most active in its category (ie, snacks), with 18 COVID-19-related posts on Facebook and Instagram (18/92, 20%) across the 4-month period.

Specific brands that were most actively posting COVID-19-related content were Domino’s and Dan Murphy’s, with both brands posting more than 100 COVID-19-related posts across the four social media platforms (Domino’s: 231/623, 37.1% of total posts; Dan Murphy’s: 119/253, 47.0% of total posts) during the 4-month period.

**Table 3:** COVID-19-related marketing on social media platforms by the top Australian unhealthy food, beverage, and alcohol brands over 4 months (February to May 2020).

Brands		COVID-19-related posts on Australian brands’ social media accounts, n/N (%)					Shares, views, and likes per COVID-19 post, mean (SD), range
		Facebook	Instagram	YouTube	Twitter	Total	
**Confectionery**							
	Cadbury^a^	10/48 (21)	3/14 (21)	N/A^b^	No posts^c^	13/62 (21)	590 (772), 14-2569
	Allen’s	1/7 (14)	N/A	0/1 (0)	N/A	1/8 (14)	43^d^
	Darrell Lea	7/50 (14)	6/44 (14)	2/7 (29)	N/A	15/101 (14.9)	6528 (19,255), 126-76,000
	Lindt	11/86 (13)	16/125 (12.8)	No posts	N/A	27/211 (12.8)	242 (269), 24-1114
	Total	29/191 (15.2)	25/183 (13.7)	2/8 (25)	—^e^	56/382 (14.7)	Not calculated^f^
**Snacks**							
	Arnott’s	10/48 (21)	8/44 (18)	No posts	No posts	18/92 (20)	554 (637), 45-1874
	Peters	0/4 (0)	N/A	N/A	N/A	0/4 (0)	—
	Magnum	0/3 (0)	N/A	No posts	N/A	0/3 (0)	—
	Smith’s	0/13 (0)	N/A	No posts	N/A	0/13 (0)	—
	Total	10/68 (15)	8/44 (1)	—	—	18/112 (16.1)	Not calculated
**Soft drinks**							
	Coca-Cola	1/6 (17)	0/4 (0)	0/1 (0)	No posts	1/11 (9)	247^d^
	Pepsi Max	2/4 (50)	N/A	0/1 (0)	N/A	2/5 (40)	29 (7), 24-34
	Schweppes	0/1 (0)	0/1 (0)	No posts	N/A	0/2 (0)	—
	Kirks^g^	N/A	N/A	N/A	N/A	N/A	—
	Total	3/11 (27)	0/5 (0)	0/2 (0)	—	3/18 (17)	Not calculated
**Quick service restaurants**							
	McDonald’s	12/23 (52)	9/20 (45)	1/13 (8)	3/3 (100)	25/59 (42)	29,387 (131,637), 33-660,977
	KFC	14/24 (58)	6/14 (43)	0/2 (0)	No posts	20/40 (50)	4061 (6136), 6-22,706
	Hungry Jack’s	17/49 (35)	9/31 (29)	1/7 (14)	4/9 (44)	31/96 (32)	3944 (16,266), 6-91,384
	Domino’s	147/365 (40.3)	70/227 (30.8)	5/7 (71)	9/24 (38)	231/623 (30.1)	1028 (3649), 2-300,015
	Subway	29/61 (48)	16/33 (48)	1/3 (33)	No posts	46/97 (47)	19,010 (65,422), 25-355,044
	Total	219/522 (42.0)	110/325 (33.8)	8/32 (25)	16/36 (44)	353/915 (38.6)	Not calculated
**Food delivery services**							
	Uber Eats	3/7 (43)	12/18 (67)	N/A	N/A	15/25 (60)	588 (557), 19-1911
	Deliveroo	0/3 (0)	7/22 (32)	No posts	0/3 (0)	7/28 (25)	819 (419), 155-1506
	DoorDash^g^	N/A	N/A	N/A	N/A	N/A	—
	Menulog	11/26 (42)	11/45 (24)	2/5 (40)	6 /13 (46)	30/89 (34)	238 (768), 0-4223
	Total	14/36 (39)	30/85 (35)	2/5 (40)	6/13 (46)	52/142 (36.6)	Not calculated
**Beer**							
	XXXX Gold	7/16 (44)	4/9 (44)	1/1 (100)	N/A	12/26 (46)	1073 (682), 265-2376
	Corona Extra	1/1 (100)	0/23 (0)	No posts	N/A	1/24 (4)	41^d^
	Carlton Premium Dry	14/23 (61)	N/A	N/A	N/A	14/23 (61)	1448 (3395), 38-12,821
	Victoria Bitter	24/62 (39)	13/52 (25)	N/A	N/A	37/114 (32.5)	1198 (660), 221-2781
	Great Northern Original Lager	6/8 (75)	14/29 (48)	2/2 (100)	N/A	22/39 (56)	19,461 (64,807), 10-282,560
	Total	52/110 (47.3)	31/113 (27.4)	3/3 (100)	—	86/226 (38.1)	Not calculated
**Spirits and ready-to-drink alcoholic beverages (combined)**							
	Smirnoff Red	0/4 (0)	No posts	N/A	N/A	0/4 (0)	—
	Johnnie Walker Red Label	No posts	N/A	No posts	N/A	No posts	—
	Jim Beam	0/14 (0)	0/4 (0)	0/2 (0)	N/A	0/20 (0)	—
	Jack Daniel’s	7/19 (37)	6/17 (35)	N/A	N/A	13/36 (36)	506 (482), 41-1546
	Bundaberg	6/13 (46)	6/11 (55)	No posts	N/A	12/24 (50)	1121 (1317), 265-5000
	Canadian Club	3/3 (100)	6/8 (75)	0/1 (0)	N/A	9/12 (75)	3044 (6427), 87-20,105
	Woodstock & Cola	0/9 (0)	0/16 (0)	N/A	N/A	0/25 (0)	—
	Total	16/62 (26)	18/56 (32)	0/3 (0)	—	34/121 (28.1)	Not calculated
**Wine**							
	De Bortoli	21/61 (34)	24/56 (43)	0/11 (0)	2/13 (15)	47/141 (33.3)	161 (154), 3-695
	McWilliam’s	7/34 (21)	7/32 (22)	N/A	N/A	14/66 (21)	39 (25), 9-84
	Stanley Wines^g^	N/A	N/A	N/A	N/A	N/A	—
	Jacob’s Creek	10/25 (40)	9/20 (45)	0/1 (0)	N/A	19/46 (41)	144 (164), 1-481
	Berri Estates	3/8 (38)	N/A	N/A	N/A	3/8 (38)	23 (23), 2-48
	Total	41/128 (32.0)	40/108 (37.0)	0/12 (0)	2/13 (15)	83/261 (31.8)	Not calculated
**Bottle shops and alcohol delivery services (combined)**							
	BWS	34/77 (44)	34/55 (68)	N/A	N/A	68/132 (51.5)	213 (376), 0-1657
	Liquorland	3/15 (20)	3/24 (13)	N/A	N/A	6/39 (15)	85 (119), 6-317
	Dan Murphy’s	75/100 (75.0)	36/135 (26.7)	4/14 (29)	4/4 (100)	119/253 (47.0)	13,751 (142,233), 2-1,552,065
	Cellarbrations	0/10 (0)	N/A	N/A	N/A	0/10 (0)	—
	IGA Plus Liquor	1/11 (9)	N/A	N/A	N/A	1/11 (9)	39^d^
	Shop My Local	1/31 (3)	0/21 (0)	N/A	N/A	1/52 (2)	3^d^
	Jimmy Brings	14/39 (36)	19/50 (38)	0/24 (0)	3/9 (33)	36/122 (29.5)	148 (360), 1-1792
	Total	128/283 (45.2)	92/285 (32.3)	4/38 (11)	7/13 (54)	231/619 (37.3)	Not calculated

^a^Cadbury represents two of the top five brands in the confectionery category: Cadbury and Cadbury Dairy Milk. Cadbury Dairy Milk was also in the top five brands within the snack category.

^b^N/A: not applicable; brand did not have an Australian social media account for this platform or had not posted since February 2019 and was excluded from analysis.

^c^Brand did have an Australian social media account for this platform, but there were no posts of any kind during this period and, therefore, no COVID-19-related posts; percentage cannot be calculated because division by zero is undefined.

^d^There was only 1 COVID-19-related post, so SD and range could not be calculated.

^e^Could not be calculated because there were no COVID-19-related posts or no posts of any kind on these platforms, the brands did not use this social media platform, or the brands did not have an Australian social media account.

^f^Grand means of shares, views, and likes per COVID-19 post within brand categories were not calculated, as there was a large variation between the brands in terms of number of followers.

^g^This brand did not have any Australian accounts and was excluded from the analysis.

The analysis of COVID-19-related posts (n=916) identified a broad range of themes used, with often more than one theme used per post, in particular when posting video content. Table 4 shows that, across all brands, the themes most consistently used were (1) *isolation activities*, which included suggestions and activities for people to do at home while in isolation (eg, cocktail recipes by BWS, online trivia nights organized by Dan Murphy’s, and virtual parties by Domino’s), and (2) *community support*.

**Table 4:** Proportion of COVID-19–related themes of social media posts by brand category.

COVID-19 theme	Posts containing each theme per category, n (%)^a^								
	Confectionery (n=56)^b^	Snacks (n=18)	Soft drinks (n=3)	QSRs^c^ (n=353)	Food delivery (n=52)	Beer (n=86)	Wine (n=83)	Spirits and RTD^d^ alcoholic beverages (n=34)	Bottle shops and alcohol delivery (n=231)
Trading and events updates	7 (13)	0 (0)	0 (0)	22 (6.2)	7 (14)	1 (1)	15 (18)	4 (12)	70 (30.3)
Home delivery and take away	2 (4)	0 (0)	0 (0)	197 (55.8)	35 (67)	1 (1)	30 (36)	0 (0)	73 (31.6)
Hygiene and contact free	2 (4)	0 (0)	0 (0)	143 (40.5)	17 (33)	0 (0)	12 (15)	4 (12)	26 (11.3)
Community support and feeling	21 (38)	4 (22)	3 (100)	56 (15.9)	19 (37)	12 (14)	24 (29)	10 (29)	7 (3.0)
Applaud health staff and essential workers	3 (5)	0 (0)	3 (100)	43 (12.2)	5 (10)	0 (0)	4 (5)	0 (0)	3 (1.3)
Donations	5 (9)	0 (0)	0 (0)	34 (9.6)	4 (8)	2 (2)	9 (11)	6 (18)	3 (1.3)
Isolation activities	25 (45)	16 (89)	2 (67)	63 (17.8)	3 (6)	48 (56)	44 (53)	14 (41)	150 (64.9)
Consumption helps coping	5 (9)	2 (11)	0 (0)	26 (7.4)	4 (8)	5 (6)	9 (11)	0 (0)	21 (9.1)
Supporting local business and trading partners	0 (0)	0 (0)	0 (0)	11 (3.1)	19 (37)	2 (2)	1 (1)	0 (0)	0 (0)
Other	0 (0)	0 (0)	0 (0)	6 (1.7)	0 (0)	11 (13)	2 (2)	2 (6)	6 (2.6)
No clear theme^e^	10 (18)	0 (0)	0 (0)	33 (9)	2 (4)	14 (16)	0 (0)	8 (24)	12 (5.2)

^a^Posts could be coded for multiple themes; therefore, the columns do not add up to 100%.

^b^All n values in this row represent total COVID-19-related posts.

^c^QSR: quick service restaurant.

^d^RTD: ready-to-drink.

^e^Indirect link to COVID-19 context (eg, using hashtags or referring to working from home).

The theme *isolation activities* was particularly prominent among alcohol brands as well as bottle shops and alcohol delivery services (combined), with 40% to 64% of all COVID-19-related posts from alcohol categories using this theme. The proportion of COVID-19-related posts that used the theme *trading and events updates* was greatest for bottle shops and alcohol delivery services (70/231, 30.3%). *Home delivery and take away*–themed posts were frequently used by bottle shops and alcohol delivery services (73/231, 31.6%) and food delivery services (35/52, 67%), but they were also used by QSRs (197/353, 55.8%) and wine brands (30/83, 36%).

**Figure 2:**
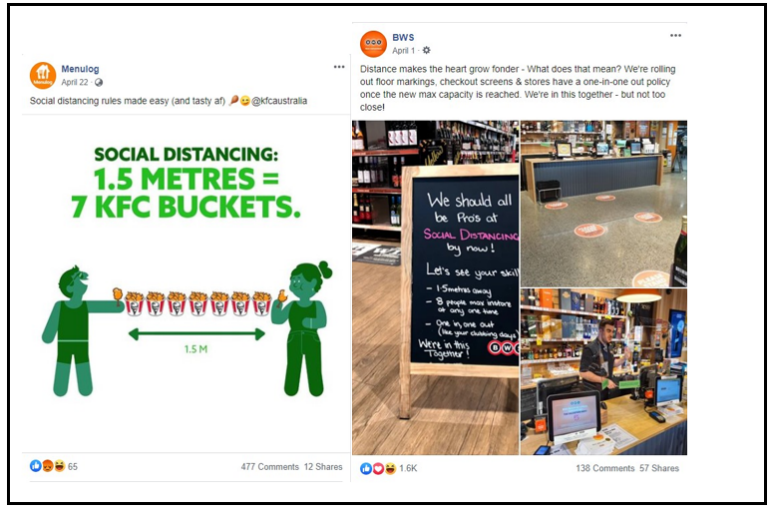
Examples of posts with the *hygiene and contact free (social distancing)* theme.

Over a third of all posts from brands representing establishments where food is handled by staff used the *hygiene and contact free* theme, such as QSRs (143/353, 40.5%) and food delivery services (17/52, 33%). This often included a mention of safe food handling practices and hygiene standards, an emphasis on contact-free delivery or pickup, and physical distancing requirements in stores (see Table 2 and Figure 2).

Brands also used the theme *applauding health staff and essential workers* for their efforts during the pandemic (QSRs: 43/353, 12.2%) or making *donations* to charity or other organizations (spirits and RTD alcoholic beverages: 6/34, 18%; wine: 9/83, 11%; QSRs: 34/353, 9.6%). Approximately 5% to 10% of COVID-19-related posts across almost every category referred to the products helping people cope with isolation by creating a sense of normality or of the consumer deserving a treat in difficult times (see Table 2).

### Food and Alcohol Parent Companies

A total of 16 out of 28 parent companies (57%) were excluded from the analysis, either because the brand and parent company used the same social media account (10/16, 63%) or because the parent company did not have a social media account (6/16, 38%). All 12 parent companies included in the analysis posted content that related to COVID-19. International parent companies such as Yum! Brands (n=194 posts), Unilever (n=133 posts), Mondelez (n=76 posts), and local Australian parent company Coca-Cola Amatil (n=70 posts) were most prolific in posting COVID-19-related content during the 4-month period under analysis. Of all COVID-19-related posts by parent companies across all social media platforms (n=783 posts), 54.7% (428/783) were on Twitter and 31.2% (244/783) were on Facebook (data not shown).

Table 5 illustrates the most frequently used themes for COVID-19-related posts by parent companies. For almost all product categories, the theme *community support* was highly prevalent, often expressed using hashtags such as #InThisTogether and #StrongerTogether. The *donations* theme was also frequently used across all categories. This could refer to financial donations to support relief organizations and hospitality staff funds or product donations, such as sanitizing products and food. For example, Mondelez posted that they donated US $20 million worth of chocolate and snacks globally, mainly to health care workers and food banks. The donations theme was often combined with other frequently used themes, namely *applauding health staff and essential workers*. For example, a Mondelez Facebook post showed a photo of health workers with Oreos and Tang—orange, powdered drink mix—in their hands (see Table 2).

**Table 5:** COVID-19–related themes of social media posts by unhealthy food, beverage, and alcohol parent companies.

COVID-19 theme	Posts containing each theme per category, n (%)^a,b^						
	Confectionery (2 companies) (n=109)^c^	Snacks (1 company) (n=133)	Soft drinks (1 company) (n=70)	QSRs^d^ (1 company) (n=194)	Beer (2 companies) (n=23)	Wine (2 companies) (n=7)	Spirits and RTD^e^ alcoholic beverages (3 companies) (n=103)
Trading and events updates	0 (0)	0 (0)	4 (6)	0 (0)	4 (17)	1 (14)	6 (5.8)
Home delivery and take away	0 (0)	0 (0)	0 (0)	27 (13.9)	0 (0)	0 (0)	0 (0)
Hygiene and contact free	1 (0.9)	8 (6.0)	5 (7)	39 (20.1)	1 (4)	0 (0)	2 (1.9)
Community support and feeling	70 (64.2)	86 (64.7)	30 (43)	114 (58.8)	17 (74)	1 (14)	20 (19.4)
Applaud health staff and essential workers	43 (39.4)	33 (24.8)	13 (19)	88 (45.4)	1 (4)	0 (0)	19 (18.4)
Donations	55 (50.5)	60 (45.1)	27 (39)	128 (66.0)	6 (26)	2 (29)	67 (65.0)
Isolation activities	11 (10.1)	6 (4.5)	5 (7)	10 (5.2)	5 (22)	0 (0)	15 (14.6)
Consumption helps coping	7 (6.4)	2 (1.5)	2 (3)	1 (0.5)	2 (9)	0 (0)	0 (0)
Supporting local business and trading partners	0 (0)	2 (1.5)	5 (7)	0 (0)	2 (9)	2 (29)	4 (3.9)
Production of sanitizing products	3 (2.8)	43 (32.3)	17 (24)	0 (0)	1 (4)	0 (0)	24 (23.3)
Health advice, including mental health	15 (13.8)	33 (24.8)	2 (3)	0 (0)	4 (17)	4 (57)	19 (18.4)
Maintaining essential supply chain	25 (22.9)	2 (1.5)	3 (4)	0 (0)	1 (4)	0 (0)	0 (0)

^a^Posts could be coded for multiple themes; therefore, the columns do not add up to 100%.

^b^The categories *bottle shops and alcohol delivery services* and *food delivery services* were excluded from this table, as there were no parent companies or no parent companies with social media accounts.

^c^All n values in this row represent total COVID-19-related posts.

^d^QSR: quick service restaurant.

^e^RTD: ready-to-drink.

Posts relating to production and supply of hand sanitizer were also frequently made by parent companies that were in the business of distilling (eg, alcohol companies) or manufacturing of sanitizing products, such as soaps and bleach. These companies also often provided *health advice* in terms of how to clean and sanitize to prevent spread of COVID-19 (see Table 2).

The prevalence of COVID-19-related posts that provided mental health advice was highest among alcohol companies, mainly through reposting videos from DrinkWise—the Australian alcohol industry’s social aspect organization—featuring a celebrity doctor warning about the use of alcohol as a coping mechanism for stress during the COVID-19 pandemic.

Some companies posted specifically to support their business or trading partners (see Table 2). Finally, 23% of confectionery companies’ posts (Mondelez and Nestlé) aimed to reassure consumers that the company was doing everything it could to maintain the “essential” food supply chain, often shown in behind-the-scenes videos of production and supply chains (see Table 2).

## Discussion

### Principal Findings

This is the first comprehensive and systematic analysis of the nature and extent of COVID-19-related online marketing by major alcohol and unhealthy food and beverage brands and their parent companies. We demonstrated that during a 4-month period (February to May 2020) of the first wave of the COVID-19 pandemic in Australia, 79% of the top 42 alcohol and unhealthy food and beverage brands as well as all 12 parent companies posted content related to COVID-19. Approximately one-third of social media posts by brands were COVID-19 related, and the number of likes, views, and shares indicate that engagement was substantial. Australian QSRs, alcohol brands, bottle shops, and food and alcohol delivery services were the most active in posting COVID-19-related marketing during this time. While brands in the snack, confectionery, and soft drink categories were relatively less active with regard to the number of COVID-19-related posts, their parent companies were often more active. COVID-19-related social media posts most commonly related to *isolation activities*, particularly for alcohol brands, with approximately half of all posts using this theme. Two other common COVID-19-related themes for both brands and companies were *community support*, positioning themselves as “in this together” with consumers, and *applauding health staff*. Parent companies also frequently posted about CSR activities, such as donations of money, food, or hand sanitizer.

Our results support and extend evidence from the marketing literature that businesses often employ cause marketing on social media platforms to improve public perception of their brand over the long term [23]. We found that parent companies, in particular, frequently posted about their CSR initiatives, such as financial donations to support relief efforts; manufacturing and/or donation of hand sanitizer by alcohol companies; donating soaps and sanitizing products, such as by the snack company Unilever; and donating chocolates, such as by the confectionery companies Mondelez and Nestlé. Communicating CSR initiatives has been shown to increase positive public attitudes to alcohol and unhealthy food brands and legitimizes their consumption [31]. Such marketing not only increases sales, but creates a fertile environment to loosen current regulations or resist further regulation of the industry, as these companies are seen as “part of the solution” [31]. For example, the industry body Alcohol Beverages Australia recently called on governments to minimize “regulatory and tax burdens” in response to the impacts of COVID-19 policies on sales [32]. Providing “health advice” in relation to avoiding contraction of COVID-19 (eg, how to wash hands) and messages related to responsible consumption of alcohol was another strategy frequently used by parent companies. While these may be viewed as health promotion messages, there is evidence to suggest that this type of messaging, when propagated by the alcohol industry, actually reinforces the idea that high levels of alcohol consumption are normal [33].

Another common reason for cause marketing is to increase immediate sales and business returns by aligning with trending topics. Our results indicate that in order to effectively engage consumers, many brands and services referred to COVID-19 in their social media posts to make themselves part of the zeitgeist, for example, by providing fun isolation activities, health and hygiene advice, and claiming “we’re all in this together.” Additionally, we found that food and alcohol delivery services, QSRs, and bottle shops responded to the rapid changes in consumer practices during the COVID-19 pandemic (eg, buying take away instead of going to restaurants). Their posts often used COVID-19 themes related to health and safety procedures, such as contact-free pickup and delivery of food and alcohol and hygienic preparation of meals. The success of this marketing and the accompanying technological advancements in online sales is illustrated by bank card data showing that Australians spent 46.2% more in bottle shops in the week ending on May 22, 2020, compared to the same week in 2019, while spending in pubs and hotels plummeted (63%) [34]. Given that the social media accounts included in our analysis each had thousands, or often hundreds of thousands, of followers, large numbers of people are likely exposed to these posts, making social media posts a very efficient form of (unpaid) advertising.

“COVID-washing” of social media posts by Big Food and Big Alcohol is propagated to ultimately further increase sales and consumption of their unhealthy products and is in direct contrast with the goal of “building back better” from COVID-19 [35]. Alcohol consumption and unhealthy food and beverage consumption are both major risk factors for excess weight gain and obesity, which in turn are risk factors for comorbidities and mortality from COVID-19 [4]. Reducing population levels of alcohol and unhealthy food consumption will not only protect against the ongoing threat from COVID-19 and other pandemics in the future but is also vital for maintaining physical and mental health during pandemics and associated times of restricted movement. Regulating the marketing of these unhealthy products is an important step toward achieving this public health goal.

### Limitations

Our study did not capture posts from smaller brands and companies. However, this is the first study to comprehensively describe the nature and extent of “COVID-washing” on social media platforms. A strength of our analysis is that we systematically captured all posts on social media platforms from the top brands (n=42) and parent companies (n=12), by market share, in Australia across a 4-month period during the COVID-19 pandemic. While examining all food and alcohol posts from social media platforms would provide a more accurate indication of COVID-19 marketing breadth, it would miss the depth that we were able to capture by focusing on the largest brands and companies. Another limitation of this study is that it only included publicly available marketing on four social media platforms and has missed posts on other social media platforms, including those where young people, who are more vulnerable to the effects of marketing, are active, such as Snapchat and TikTok. Our initial scoping of posts by the brands and companies included in our study suggested that the brands and companies under analysis did not have public accounts on these social media platforms; however, further work is required to better understand how these social media platforms are used by alcohol and food companies to engage consumers, including during times of crisis. A further limitation is that we were unable to determine from our analysis what the reach and actual exposure levels to these posts were. This information is only available to the marketers. However, the number of followers of active brand accounts and the level of engagement with COVID-19-related posts suggest that reach was quite high. Finally, this study only captured a portion of the marketing material created by these brands. We included public posts on official brand and company accounts, but not user-generated content, which is now an integral part of brands’ and companies’ marketing campaigns (eg, the use of influencers). We also did not capture paid advertisements, as companies are unlikely to post ads on their public pages. Thus, the practices we have identified may well be much more extensive and culturally embedded than what we describe here. It is important that future studies are designed to monitor both covert and overt marketing techniques.

### Conclusions

“COVID-washing” by Big Food and Big Alcohol is both common and concerning. Brands and companies have strategically designed their marketing campaigns to positively align with the COVID-19 pandemic to increase brand loyalty and sales and potentially to influence and oppose regulation. This is problematic, as consumption of alcohol and unhealthy foods are directly or indirectly associated with poorer COVID-19-related outcomes; they also negatively impact physical and mental health beyond the pandemic. The implementation of comprehensive regulations to restrict unhealthy food and alcohol marketing, as recommended by the World Health Organization [36,37], is particularly acute in the COVID-19 context and is urgently required to “build back better” in a post-COVID-19 world.

## Appendix

Multimedia Appendix 1 Inclusion of brands and parent companies in the audit.

## Abbreviations

CSR: corporate social responsibility
QSR: quick service restaurant
RTD: ready-to-drink
VicHealth: Victorian Health Promotion Foundation
