# Supplementary material for: The Nature and Extent of Online Marketing by Big Food and Big Alcohol During the COVID-19 Pandemic in Australia: Content Analysis Study
Source: JMIR Public Health Surveill. 2021 Mar 12;7(3):e25202. doi: 10.2196/25202 (PMC7958974; doi:10.2196/25202)
Supplement: Multimedia Appendix 1 [file publichealth_v7i3e25202_app1.docx]

**Multimedia Appendix 1. Brands’ and parent companies’ inclusion in audit**

| **Brand** | **Included/Excluded in audit** | **Parent Company** | **Included/Excluded in audit** | **Categories** |
| --- | --- | --- | --- | --- |
| Cadbury Dairy Milk | Included as ‘Cadbury’ | Mondelez | Included | Confectionery |
| Cadbury | Included | Mondelez | As per above | Confectionery |
| Allen's | Included | Nestlé Australia Ltd | Included | Confectionery |
| Darrell Lea | Included | Darrell Lea Chocolate Co Pty Ltd | Excluded, same platforms as brand | Confectionery |
| Lindor | Included | Lindt & Sprüngli (Australia) Pty Ltd | Excluded, same platforms as brand | Confectionery |
| Arnott's | Included | Arnott's Biscuits Ltd | Excluded, same platforms as brand | Snacks |
| Peters | Included | Froneri Ltd | Excluded, no social media platforms | Snacks |
| Smith's | Included | Smiths Snackfoods Co Pty Ltd, The | Excluded, same platforms as brand | Snacks |
| Cadbury Dairy Milk | Included as ‘Cadbury’ | Mondelez | As per above | Snacks |
| Magnum | Included | Unilever Australia Ltd | Included | Snacks |
| Coca Cola | Included | Coca-Cola Amatil Ltd | Included | Soft drink |
| Pepsi Max | Included | Schweppes Australia Pty Ltd | Excluded, same platforms as brand | Soft drink |
| Coca-Cola No Sugar | Included as ‘Coca Cola’ | Coca-Cola Amatil Ltd | As per above | Soft drink |
| Schweppes | Included | Schweppes Australia Pty Ltd | As per above | Soft drink |
| Kirks | Excluded, no social media presence >12 months | Coca-Cola Amatil Ltd | As per above | Soft drink |
| McDonald's | Included | McDonald's Australia Ltd | Excluded, same platforms as brand | Chained consumer foodservice |
| KFC | Included | Yum! Brands Inc (international) | Included | Chained consumer foodservice |
| Hungry Jack's | Included | Restaurant Brands International Inc | Excluded, no social media platforms | Chained consumer foodservice |
| Domino's Pizza | Included | Domino's Pizza Enterprises Ltd | Excluded, same platforms as brand | Chained consumer foodservice |
| Subway | Included | Subway Systems Australia Pty Ltd | Excluded, same platforms as brand | Chained consumer foodservice |
| Uber Eats | Included | Uber Eats | Excluded, same platforms as brand | Food delivery service |
| Deliveroo | Included | Deliveroo | Excluded, same platforms as brand | Food delivery service |
| DoorDash | Excluded, no Australian social media accounts | DoorDash | Excluded, same platforms as brand | Food delivery service |
| Menulog | Included | Menulog | Excluded, same platforms as brand | Food delivery service |
| XXXX Gold | Included | Lion Pty Ltd | Included | Beer brands |
| Corona Extra | Included | Carlton & United Breweries (CUB) | Included | Beer brands |
| Carlton Premium Dry | Included | Carlton & United Breweries (CUB) | As per above | Beer brands |
| Victoria Bitter | Included | Carlton & United Breweries (CUB) | As per above | Beer brands |
| Great Northern Original Lager | Included | Carlton & United Breweries (CUB) | As per above | Beer brands |
| Smirnoff Red | Included | Diageo Australia Ltd | Included | Spirits |
| Johnnie Walker Red Label | Included | Diageo Australia Ltd | As per above | Spirits |
| Jim Beam | Included | Beam Suntory (international) | Included | Spirits |
| Jack Daniel's | Included | Brown-Forman (international) | Included | Spirits |
| Bundaberg | Included | Diageo Australia Ltd | As per above | Spirits |
| Canadian Club | Included | Beam Suntory (international) | As per above | RTDs |
| Jim Beam Cola | Included as ‘Jim Beam’ | Beam Suntory (international) | As per above | RTDs |
| Jack Daniel's Whiskey & Cola | Included as ‘Jack Daniel's’ | Brown-Forman (international) | As per above | RTDs |
| Bundaberg Rum & Cola | Included as ‘Bundaberg’ | Diageo Australia Ltd | As per above | RTDs |
| Woodstock & Cola | Included | Asahi Premium Beverages Pty Ltd | Excluded, no social media platforms | RTDs |
| De Bortoli | Included | De Bortoli Wines Pty Ltd | Excluded, same platforms as brand | Wine |
| McWilliam's | Included | McWilliam's Wines Pty Ltd | Excluded, same platforms as brand | Wine |
| Stanley Wines | Excluded, no social media platforms | Accolade Wines Australia Ltd | Included | Wine |
| Jacob's Creek | Included | Pernod Ricard Winemakers Pty Ltd | Included | Wine |
| Berri Estates | Included | Accolade Wines Australia Ltd | Included | Wine |
| BWS | Included | Woolworths Group Ltd | Excluded, no social media platforms | Bottle shops, alcohol delivery service |
| Liquorland | Included | Coles Group | Excluded, no social media platforms | Bottle shops |
| Dan Murphy's | Included | Woolworths Group Ltd | As per above | Bottle shops, alcohol delivery service |
| Cellarbrations | Included | MetCash | Excluded, no social media platforms | Bottle shops |
| IGA Plus Liquor | Included | MetCash | As per above | Bottle shops |
| Jimmy Brings | Included | Jimmy Brings | Excluded, same platforms as brand | Alcohol delivery service |
| Shop my Local | Included | Independent Brands Australia | Excluded, no social media platforms | Alcohol delivery service |

*Source: Euromonitor, 2019*
